# Orbital Cystic Schwannoma Originating from the Frontal Nerve

**DOI:** 10.1155/2012/604574

**Published:** 2012-12-23

**Authors:** Yasuhiko Hayashi, Takuya Watanabe, Daisuke Kita, Yutaka Hayashi, Masayuki Takahira, Jun-ichiro Hamada

**Affiliations:** ^1^Department of Neurosurgery, Graduate School of Medical Science, Kanazawa University, 13-1 Takara-machi, Kanazawa 920-8640, Japan; ^2^Department of Ophthalmology, Graduate School of Medical Science, Kanazawa University, Kanazawa 920-8641, Japan

## Abstract

Schwannomas of the orbit are very rare benign neoplasms. Intraorbital cystic schwannomas originating from the frontal nerve are even rarer, with only 1 case reported to date. This is most likely due to the fact that, in most cases, the origin of the orbital schwannoma cannot be identified intraoperatively. The nerve origin is usually speculated from histological examination of the specimen and the postoperative neurological deficits of the patient. Here, we present the case of a 65-year-old woman with a one-month history of exophthalmos, whose orbital cystic lesion was completely removed by microsurgical transcranial operation. Intraoperatively, the continuity between the tumor and frontal nerve was seen macroscopically, leading us to confirm the frontal nerve as an origin of the tumor, which was consistent with the postoperative neurological findings. The diagnosis of the tumor was established as schwannoma from the histological examination. As a differential diagnosis of the orbital cystic lesions, the possibility of schwannomas should be kept in mind.

## 1. Introduction

Schwannoma of the orbit accounts for 1–4% of all orbital neoplasms [1–3]. Cystic lesions, such as dermoid cysts or mucoceles, of the orbit are often encountered, but cystic schwannomas are extremely rare [4–6]. Further, cystic schwannoma originating from the frontal nerve is even rarer, and only 1 case has been reported [7]. The origin of orbital tumor usually could not be found intraoperatively, because of the distal portion of the nerve derived from the tumor embedded into the adipose tissue making it difficult to discern the tumor from the original nerve [1, 8]. Here, we present the case of a 65-year-old woman with a one-month history of exophthalmos; the causative orbital cystic lesion was completely removed by microsurgical transcranial operation leaving faint ipsilateral forehead numbness. The histological diagnosis was ascertained that the tumor was schwannoma and macroscopic intraoperative finding indicated the frontal nerve as the tumor origin.

## 2. Case Report

**Figure 1:**
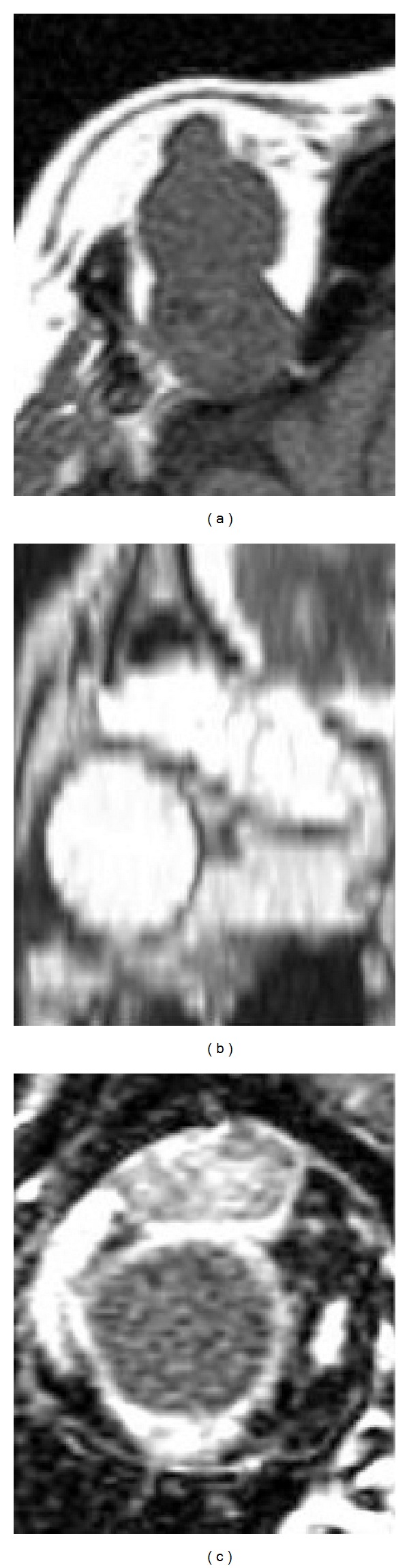
Axial magnetic resonance imaging (MRI) revealed that the lesion was located in the extraconal region, just above the levator palpebral muscle; the mass was found to be isointense on T1-weighted image (a) and to have hyperintensity on T2-weighted image (b). (c) Coronal MR image after fat suppression demonstrated marginal enhancement of the cystic mass.

 A 65-year-old woman presented with a one-month history of the right-sided exophthalmos and was referred to our hospital. On neurological examination, we found that both her pupillary size and reaction to light were normal, and her eye ball movement was not restricted. She did not have numbness in her forehead nor did she have skin lesions suggesting neurofibromatosis. Computed tomography showed a round mass in her right orbit. Magnetic resonance imaging (MRI) was performed; T1-weighted image revealed that the mass was isointense, and T2-weighted image showed that it was hyperintense (Figures 1(a) and 1(b)). A dynamic study using gadrinium-diethylene triamine pentaacetic acid on the fat suppressed T1-weighted image demonstrated the marginal enhancement of the mass (Figure 1(c)), which made us consider the mass was consisted of a cystic tumor. The mass was located in the extraconal region, just above the levator palpebrae muscle, and measured 3.0 × 2.0 × 2.0 cm. Digital subtraction cerebral angiography showed no vascular abnormalities related to the tumor.

**Figure 2:**
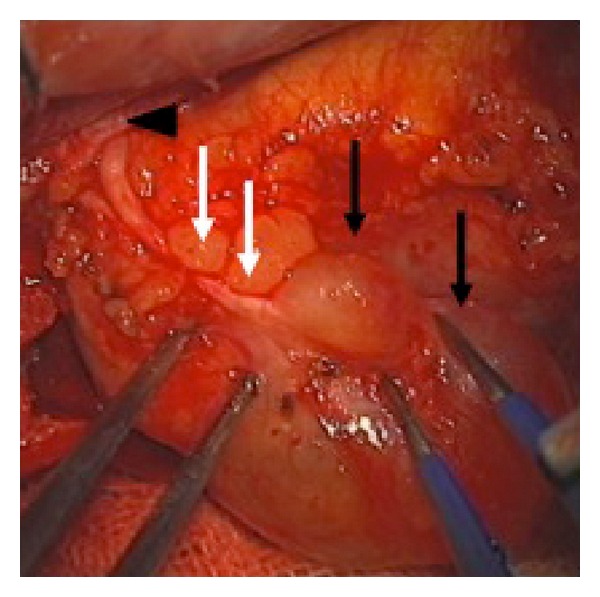
Photograph of the surgical field showed the continuity between the tumor (black arrows) and the frontal nerve (white arrows). The finding that the nerve ran toward the supraorbital foramen (arrow head) led to the conclusion of the frontal nerve being the origin of this tumor.

 The patient underwent surgical removal of the orbital tumor in our hospital. Upon right frontal craniotomy with orbital unroofing, the orbital cystic mass appeared to be partially embedded in the adipose tissue without an adhesion to the levator muscle, confirming its location in the extraconal space. Therefore, the tumor could be completely dissected from the surrounding tissues. The cyst capsule was preserved in an unruptured form, and the proximal part of the frontal nerve was confirmed to have been embedded into the tumor capsule entirely (Figure 2). After removal of the entire tumor, the levator palpebrae superioris muscle was exposed. 

**Figure 3:**
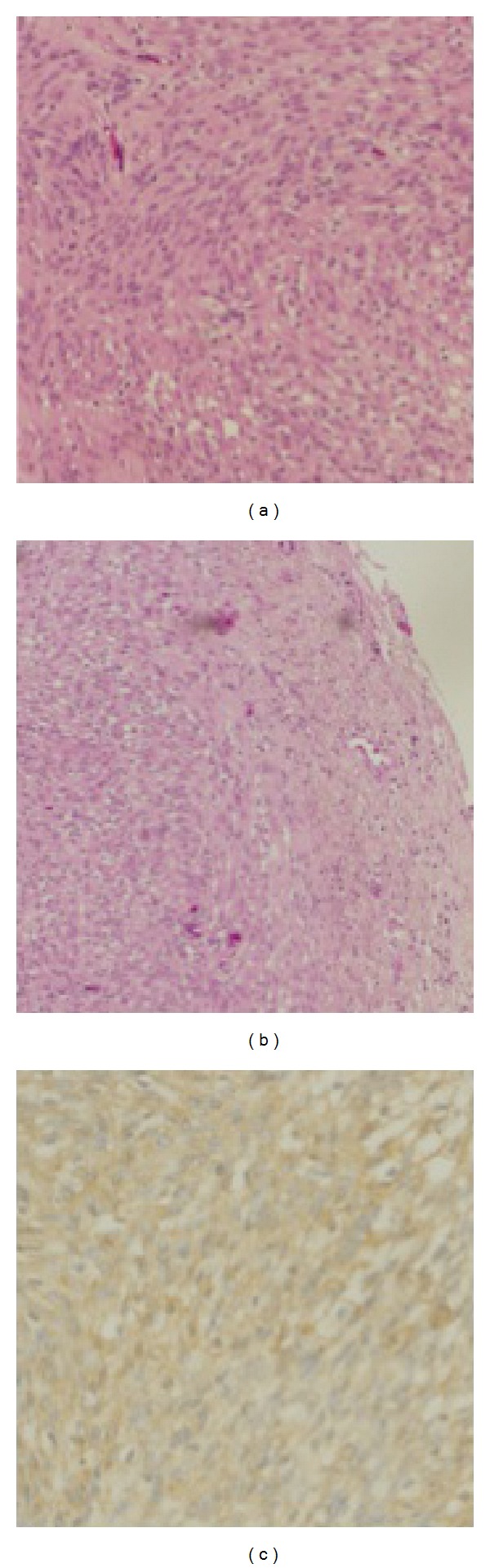
(a) Photomicrograph of the tumor showing both Antoni A type tumor cells (left, consisting of dense spindle-shaped cells with nuclear palisading nuclei) and Antoni B type tumor cells (right, sparse oval cells with phlegmatic interstitium) (hematoxylin and eosin, ×100). (b) The tumor cells were surrounded by dense connective tissue (hematoxylin and eosin, ×40). (c) An immunohistochemical examination showed that the tumor cells were strongly positive for vimentin (×100).

 Histological examination established the diagnosis of the tumor as schwannoma, and both Antoni A and B type tumor cells were noted (Figure 3(a)). The cyst of the tumor was surrounded by thick capsule and Antoni B type tumor cells were presented adjacent to the connective tissue of this capsule (Figure 3(b)). An immunohistological examination showed that the tumor cells were strongly positive for vimentin (Figure 3(c)) and S-100 (data not shown).

 Postoperative MRI confirmed the complete removal of the tumor from the orbital extraconal space. The patient was discharged with mild restriction of upward eye movement, palpebral ptosis, and numbness in the right forehead. One year after the operation, the numbness of the forehead persisted, but no recurrence of tumor was observed.

## 3. Discussion

 Although cystic lesions of the orbit are often encountered, they are most often dermoid cysts and/or mucoceles [3, 4, 9]; cystic schwannomas are extremely rare [2, 4, 6, 10]. Only 3 cases of cystic schwannomas have been previously reported [7]; In 2 of these cases, the tumors were located in the intraconal region and originated from the inferior division of the oculomotor nerve. In the other case, the tumor was located in the extraconal region with the first division of trigeminal nerve as the origin (frontal nerve) [7, 11].

 Schwannomas usually arise from Schwann cells and grow along cranial, peripheral, and autosomic nerves [11]. They predominantly stem from sensory nerves rather than motor nerves. About 24% of orbital schwannomas have been reported to originate from the first division of the trigeminal nerve; however, the origin of more than half of the orbital schwannomas could not be identified from preoperative radiological findings, intraoperative anatomical findings, and/or clinical manifestations. Therefore, postoperative neurological deficits, such as limitation of eyeball movement and numbness of the forehead, usually led neurosurgeons to identify the oculomotor nerves and trigeminal nerve as the origins of these tumors [1, 6]. Our case is the second case to exhibit a cystic schwannoma originating from the frontal nerve; the diagnosis of the tumor origin in the first reported case was merely based on the postoperative symptom and not on the intraoperative findings as demonstrated in our case [7].

 In previous cases, it was very difficult to determine the origin of the orbital schwannoma during the operation, because the distal portions of the nerve were so thinned and fanned out that the continuity between the nerves and the tumor could hardly be recognized [1, 8]. However, in our patient, the distal portion of the nerve was confirmed to have reached and passed through the ipsilateral supraorbital foramen. Therefore, the nerve could readily be identified as the frontal nerve. 

 In addition, the postoperative presenting symptom of numbness in the right half of the forehead persisted until the last visit to our outpatient clinic. Pathological microscopic findings showed the tumor to be a schwannoma. The immunohistological findings confirmed this diagnosis as the tumor cells were positive for S-100 and vimentin, both of which are known to be present in schwannoma. 

 The best treatment for orbital schwannomas is total surgical excision without complication [8]. The surgical approach would be dependent on the location of the tumors. Intraorbital schwannomas predominantly exist in the upper part of both the intracone and extracone [4, 6, 12]; therefore, anterior orbitotomy or supra orbitotomy is preferred in many cases. However, lateral and transcranial orbitotomy should also be included in the options, if a wide operative view is necessary for safe and sufficient dissection.

 The exact developmental mechanism of cystic degeneration for this type of tumor remains unknown and there is no report in the literature demonstrating a histological change. Cystic areas are usually secondary to the coalescence of mucinous or microcystic regions in Antoni B tissues of the schwannoma. Speculative mechanisms include an insufficient vascular supply resulting in necrosis and cystic changes, hemorrhage into the tumor with blood resorption causing cyst formation, and hyaline degeneration leading to cyst formation [5, 13, 14]. As shown in our case, although orbital schwannomas with a cystic transformation are certainly rare, neurosurgeons need to include cystic schwannoma in the list of conditions considered during the differential diagnosis of preoperative orbital or periorbital cystic masses.

## Abbreviation

MRI: Magnetic resonance imaging.
